# Synthesis and Characterization of Fullerene Nanowhiskers by Liquid-Liquid Interfacial Precipitation: Influence of C_60_ Solubility

**DOI:** 10.3390/molecules17043858

**Published:** 2012-03-29

**Authors:** Marappan Sathish, Kun’ichi Miyazawa

**Affiliations:** 1 Institute of Multidisciplinary Research for Advanced Materials, Tohoku University, Katahira 2-1-1, Aoba-ku, Sendai, 980-8577, Japan; 2 Fullerene Engineering Group, Materials Processing Unit, National Institute for Materials Science, 1-1, Namiki, Tsukuba, Ibaraki, 305-0044, Japan

**Keywords:** fullerene nanowhiskers, polymerization, liquid-liquid interface, C_60_ solubility, fullerene

## Abstract

Fullerene nanowhiskers (FNWs) composed of C_60_ fullerene molecules were prepared using the liquid–liquid interfacial precipitation (LLIP) method in the carbon-disulfide (CS_2_) and isopropyl alcohol (IPA) system. The electron microscopic images reveal the formation of non-tubular FNWs. The X-ray diffraction (XRD) pattern studies indicate the presence of fcc crystalline structure and unusual triclinic structure in the FNWs. The selected area electron diffraction pattern (SAED) analysis demonstrates the existence of triclinic and electron beam assisted fcc to tetragonal crystalline phase transformation. The formation of triclinic structure might be validated due to the partial polymerization of FNWs at C_60_ saturated CS_2_-IPA interface. The high solubility of C_60_ in CS_2_ solvent system results in partial polymerization of FNWs. The polymerization of fullerene molecules in the FNWs has been further confirmed using Raman spectroscopy.

## 1. Introduction

Fullerenes have created great interest in material chemistry owing to their peculiar structure and properties. Recent research has been focused on the preparation of fullerene nanostructures with fullerenes or substituted fullerene derivatives. Recently, various structural morphologies have been reported for fullerene and fullerene derivatives in the solid state as well as in solution [1,2]. In addition to the solvated structures in various solvents, the morphologies like whiskers, nanotubes, fibers, disks, cones, *etc*., have been successfully obtained by various preparation methods [3,4]. Among them, FNWs prepared using the liquid-liquid interfacial precipitation method is the most investigated in the literature [5]. Further, there are a few other reported methods for the preparation of FNWs, including the template synthesis method [6,7]. However, the liquid-liquid interfacial method for the synthesis of fullerene nanostructures is quite simple and convenient. In our earlier studies, we have shown the LLIP to be the most effective preparation procedure for nanoporous FNWs, metal ion incorporated nanowhiskers and size tunable fullerene nanosheets by changing the solvent system [8,9,10,11]. The nature of the solvent used at the interface plays major role on the morphology and structure of the fullerene nanostructure. Similarly, the solubility of the fullerene molecules in different solvents varies significantly at room temperature and pressure [12]. It is speculated that the degree of solubility of fullerene may alter the liquid-liquid interface properties, and consequently, the morphology and structure of the resulting fullerene nanostructures. 

Generally, polymerization of fullerene will occur at high pressure and high temperature treatment [13,14]. Also, photo-assisted polymerization of fullerenes is well known in literature [15,16,17]. Thus, it is clear that additional energy is required for the polymerization of fullerene molecules. In the LLIP method, there will be an interfacial pressure due to: (i) pressure difference at the liquid-liquid interface owing to the different viscosities of the liquids; (ii) Laplace pressure caused by the interfacial tension between the liquids [18]. Increasing fullerene solubility in one of the solvents will increase the liquid-liquid interfacial pressure. The high solubility of fullerene in CS_2_ is expected to increase the liquid-liquid interfacial pressure enormously. It is speculated that this interfacial pressure is sufficient for the polymerization of FNWs. This can also account for the fact that such a polymerization is indeed not observed with other solvents systems. Thus, the peculiarity of CS_2_-IPA LLIP method arises from the high interfacial pressures (due to high solubility of C_60_ in CS_2_) created at the C_60_ saturated CS_2_-IPA interface. Although, the laser or UV light assisted polymerization of fullerene molecules have been reported in the FNWs prepared at the liquid-liquid interfacial precipitation method, so far there are no reports on fullerene polymerization at the liquid-liquid interface without any external pressure or photo-irradiation. In the present study, a direct evidence for the polymerization of fullerene molecules in the FNWs is observed from the formation of triclinic crystal system.

## 2. Results and Discussion

Optical microscopy studies of the FNWs revealed the formation of long C_60_ whiskers that can be obtained only without disturbing the interface or by gentle mixing of the interface before storing the bottles in the incubator. Vigorous mixing or ultrasonication caused severe damage to the FNWs and results only in fullerene precipitates. This observation hints that a high interfacial pressure exists between the two liquids, which increases the nucleation rate when additional energy was supplied by ultrasonication or shaking. 

**Figure 1:**
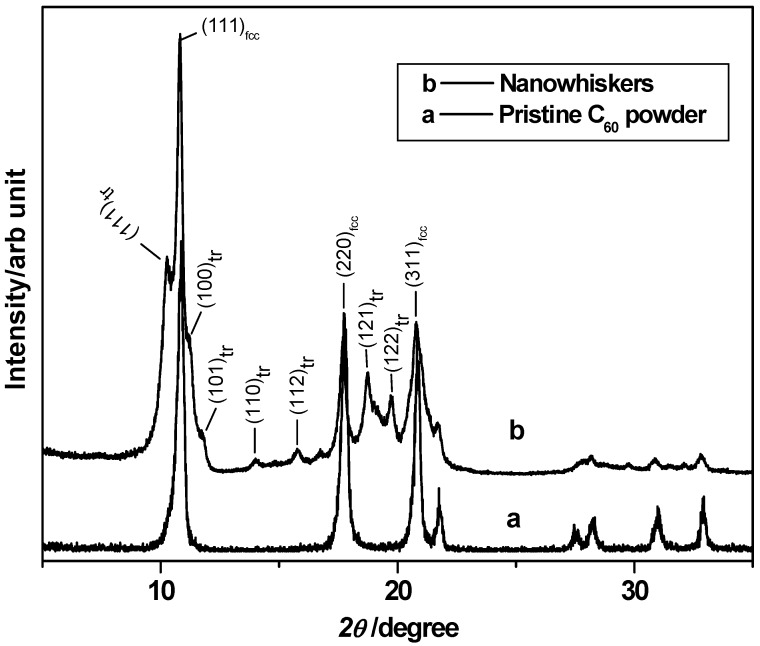
XRD pattern of (**a**) pristine C_60_ powder (**b**) nanowhiskers.

The crystalline structure of the obtained FNWs after 24 h incubation was characterized using XRD with CuK_α_ radiation. The observed pattern was compared with pristine C_60_ powder (Figure 1) and the calculated lattice constant a = 1.416 ± 0.003 nm of the FNWs reveals the presence of fcc crystalline nature. However, there are a few additional lines, which could be indexed by assuming the triclinic phase of FNWs [19]. The calculated lattice constants are a = 1.033(2) nm, b = 0.971(1) nm, c = 1.127(3) nm, α = 53.7°, β = 52.3°, γ = 58.3° and the calculated primitive unit cell volume is 0.696 nm^3^. Generally, the triclinic crystalline phase could be seen in the polymerized fullerene samples. It is speculated that polymerization of fullerene molecules in the FNWs might be taking place due to the interfacial forces generated at the C_60_ saturated CS_2_ and IPA interface. Since the solubility of fullerene in CS_2 _is high (7.9 mg/mL), the interfacial forces generated at the interface are sufficient for the polymerization. In this case, though the calculated lattice parameters are in good agreement with the reported triclinic phase, the cell volume 0.696 nm^3^ is higher than that of the reported triclinic structure [19]. Indeed, it is very close to the cell volume of fcc crystalline structure of 0.710 nm^3^. When low solubility solvents of fullerene such as benzene (1.7 mg/mL) and toluene (2.8 mg/mL) were used with IPA solution, only fcc crystalline structure was observed [8,10], and a slightly polymerized phase of fullerene was observed during the Raman spectroscopic measurements owing to the laser light assisted polymerization [8,10]. However, in CS_2_-IPA interface a clear polymerized phase could be seen in the as prepared FNWs. Therefore, a partial polymerization in the FNWs might be possible owing to the high degree of interfacial forces generated, which results the formation of unusual crystalline structure under the conventional experimental conditions. 

**Figure 2:**
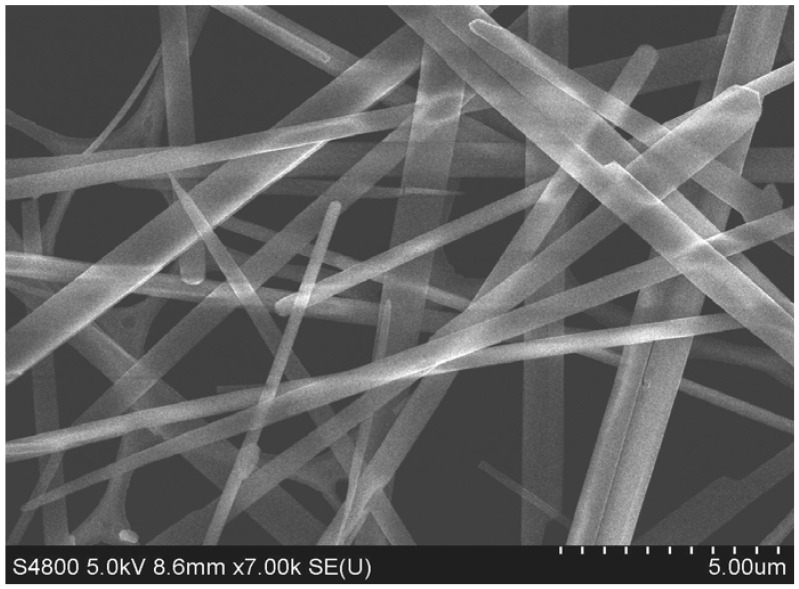
SEM image of C_60_ nanowhiskers.

The field emission scanning electron microscopy (FE-SEM) image of FNWs is shown in Figure 2. The highly smooth and uniform nature of the FNWs surface is apparently visible from the image. The calculated diameter of the FNWs varies between 300 nm to 1 μm and the length of FNWs is a few tens in micrometers. 

**Figure 3:**
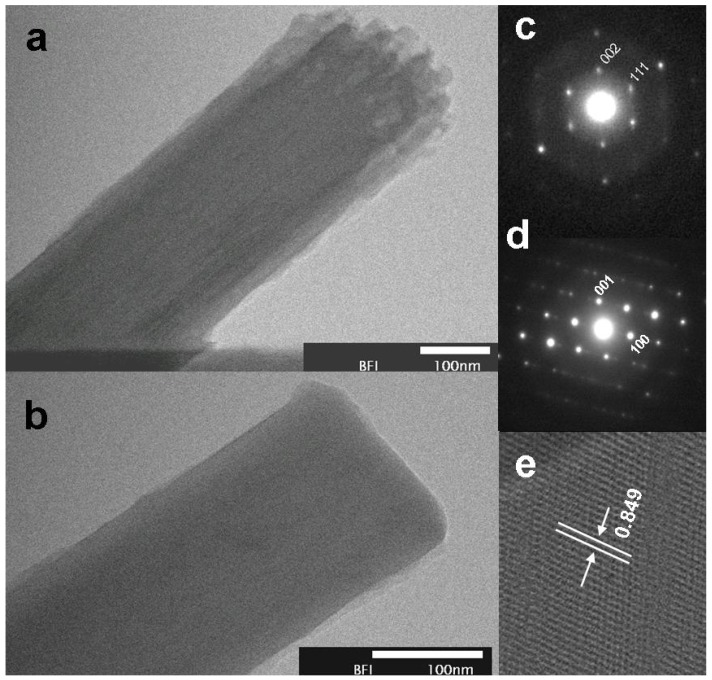
STEM images of FNWs (**a & b**), electron diffraction patterns (**c & d**) and lattice fringing (**e**) of FNWs prepared at CS_2_-IPA interface.

TEM images in Figure 3a,b clearly show the formation of non-tubular FNWs. In our earlier studies, we have reported the formation of porous tubular and non-tubular FNWs in benzene [8], whereas in the present case, there were no pores and formation of tubular FNWs were observed. This clearly shows that the nature of the solvent plays a critical role on the formation and morphology of the FNWs. In the present study, two different types of electron diffraction patterns were observed as expected from the XRD results. Figure 3 shows the electron diffraction pattern of the FNWs revealing a tetragonal structure (Figure 3c) and a triclinic crystalline structure (Figure 3d). The XRD results failed to reveal the presence of this tetragonal structure, and instead indicate the presence of an fcc structure. 

This phenomenon can be attributed to an electron beam assisted polymerization, wherein the fcc structure of the FNWs gets converted to a tetragonal phase by the impingement of the electron beam over the FNWs. The studies on laser light mediated polymerization of fullerene nanostructures support the above conclusion [16,17]. The calculated lattice parameters for the tetragonal crystals are a = 1.295 nm and c = 1.54 nm, which is in close concurrence with the tetragonal two-dimensional polymers of C_60_ [20,21]. However, the electron diffraction pattern in Figure 3d confirms the presence of a triclinic crystalline structure. Besides, a clear lattice fringing (Figure 3e) has been observed with a lattice plane spacing of 0.849 nm, corresponding to the *d *value of (111) plane for triclinic system. The combination of XRD and SAED pattern analysis makes clear that the as-prepared FNWs contain both fcc and partially polymerized triclinic structure. And, the fcc structures converted to tetragonal structure by the electron beam during the TEM-SAED measurements.

**Figure 4:**
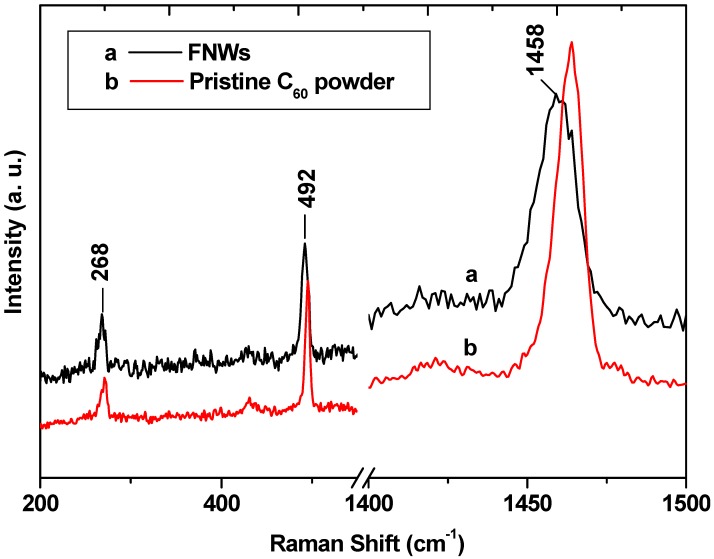
Raman spectroscopy of (**a**) FNWs and (**b**) pristine C_60_ powder.

Raman spectroscopic profiles (Figure 4) of the prepared FNWs and pristine C_60_ powders show several active lines. Among the Raman active lines, the most significant one is the line at 1469 cm^−1^, which corresponds to the ‘pentagonal pinch’ mode or A_g_(2) mode, that has been used extensively as an analytical probe for the structural and electronic properties of C_60_ molecules [22]. This mode is very susceptible to intermolecular bonding. The prepared FNWs nanowhiskers show a very clear downshift for the A_g_(2) mode compared to pristine C_60_ powder. Pristine C_60_ molecules show a strong line at 1466 cm^−1^ corresponds to Ag(2) mode (or) “pentagonal pinch” mode [23]. While, a noticeable peak shift for the Ag(2) mode at 1458 cm^−1^ was observed for the FNWs, ~8 cm^−1^ downshift compared to the pristine C_60_ powder. The observed downshift from 1469 cm^−1^ can be attributed to the polymerization of C_60_ molecules in the FNWs [23]. However, similar observation in the downshift has been observed for the photo-assisted polymerization of C_60_ solid [15,24]. Hence, it is essential to confirm the origin of the polymerization process, *i.e.*, the polymerization is due to interfacial force or photo-assisted polymerization by the laser beam, which was used for the Raman spectroscopic measurements. The observed triclinic crystalline structures with higher cell volume indicates that the interfacial forces generated at the liquid-liquid interface are may not be strong enough to completely polymerize the fullerene molecules. Thus, a partial polymerization by the weak interfacial forces results polymerization with high cell volume. From the above observations, it is believed that partial or weak polymerization of fullerene molecules in the FNWs occurs during the preparation itself due to the interfacial forces. The laser light used for the Raman spectroscopic measurements may further enhance it.

The yield of the FNWs prepared in the CS_2_ and IPA solvent system was calculated and the results showed nearly 70 wt% yield of FNWs at 5 °C. The high FNW yield can be due to the nature of the solvent, the ratio between the two solvents and the temperature. Additionally, the higher solubility of fullerene in CS_2_ might be facilitating the formation of FNWs in higher yields.

## 3. Experimental

### General

In the present study, FNWs have been prepared by forming interface between C_60_ saturated CS_2_ and IPA at 5 °C. For a typical preparation, C_60_ saturated CS_2_ solution (1 mL) was placed in a thoroughly cleaned 50 mL glass bottle and cooled to 5 °C in an ice water bath. Next IPA (4 mL) was slowly added into the C_60 _solution, the temperature was maintained at 5 °C using ice bath during the addition. The resulting mixture was stored at 5 °C in an incubator with transparent plastic window for 24 h at ambient pressure (1 atm) to grow FNWs.

Powder X-ray diffraction patterns were recorded in a RIGAKU (RINT2000 Tokyo, Japan) diffractometer using Ni-filtered Cu-K_α_ radiation (λ = 1.5418 Å). The morphology of the prepared whiskers was observed using a Hitachi-4800 FE-SEM. Scanning and high-resolution transmission electron micrographs were recorded with a JEOL JEM-2100F microscope, working at an accelerating voltage of 200 kV. Raman spectra were recorded using a micro-Raman system NRS-3100 (JASCO, Japan) spectrophotometer equipped with a semiconducting laser at a wavelength of 532 nm. 

## 4. Conclusions

Partially polymerized FNWs have been prepared by the LLIP method using a C_60_ saturated CS_2_ and IPA solvent system. The XRD study confirms the presence of fcc and triclinic crystalline structures in the as-prepared FNWs. Electron microscopic studies reveal the formation of non-tubular FNWs and the SAED pattern analysis show the electron beam assisted structural transformation of fcc to tetragonal crystalline structure. The observed triclinic crystalline phase in XRD and electron diffraction analysis, and Raman spectroscopic studies evidently confirm the polymerization of FNWs. The high solubility of fullerene in CS_2_ is expected to increase the liquid-liquid interfacial pressure enormously and it is sufficient for the polymerization of FNWs, therefore a partial polymerization in the FNWs might be possible due to the high degree of interfacial forces generated, which results the formation of unusual crystalline structures under the conventional experimental conditions.
